# Supplementary material for: Tracing the Origin of the RSPO2 Long-Hair Allele and Epistatic Interaction between FGF5 and RSPO2 in Sapsaree Dog
Source: Genes (Basel). 2022 Jan 1;13(1):102. doi: 10.3390/genes13010102 (PMC8775186; doi:10.3390/genes13010102)
Supplement: Supplementary file 1 [file genes-13-00102-s001.zip › Supplementary Figures.pdf]

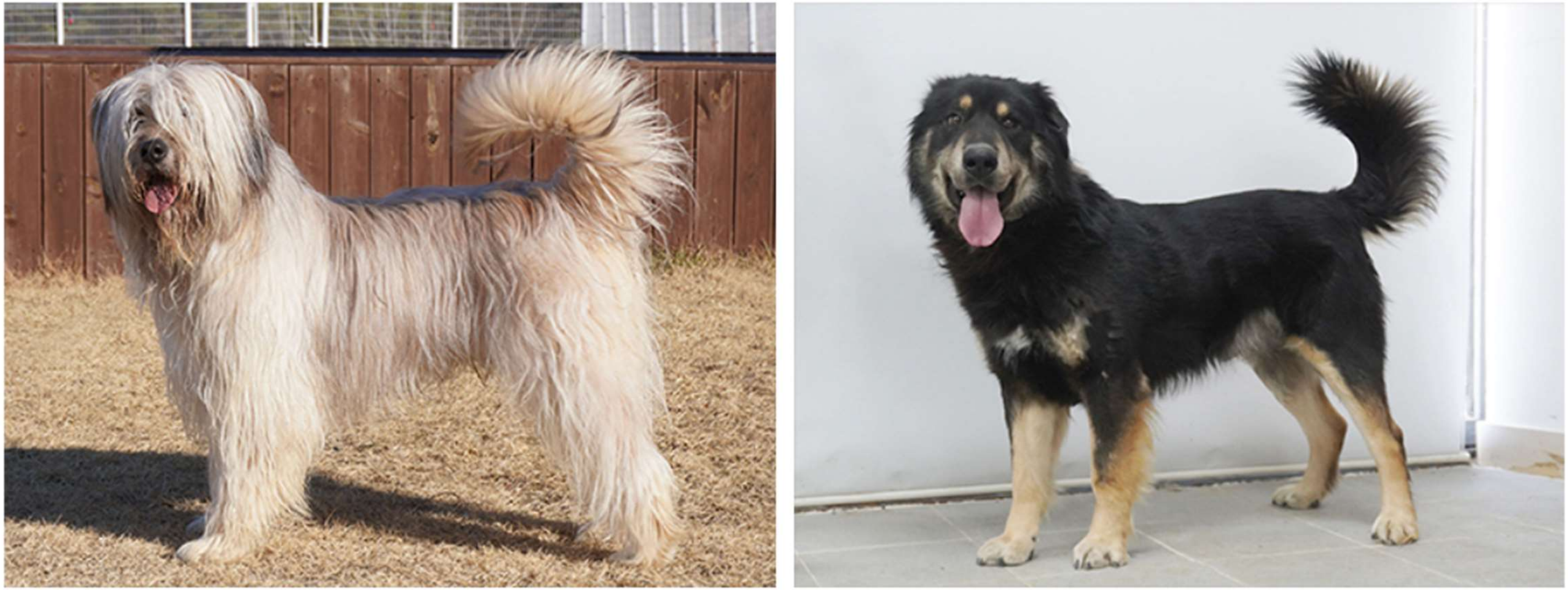

Figure S1. Pictures of native Korean Sapsaree dogs with different hair length phenotypes. In the picture, from left to right, are long- and short-haired Sapsaree dogs. Pictures were from the Korean Sapsaree foundation (http://eng.sapsaree.org/).

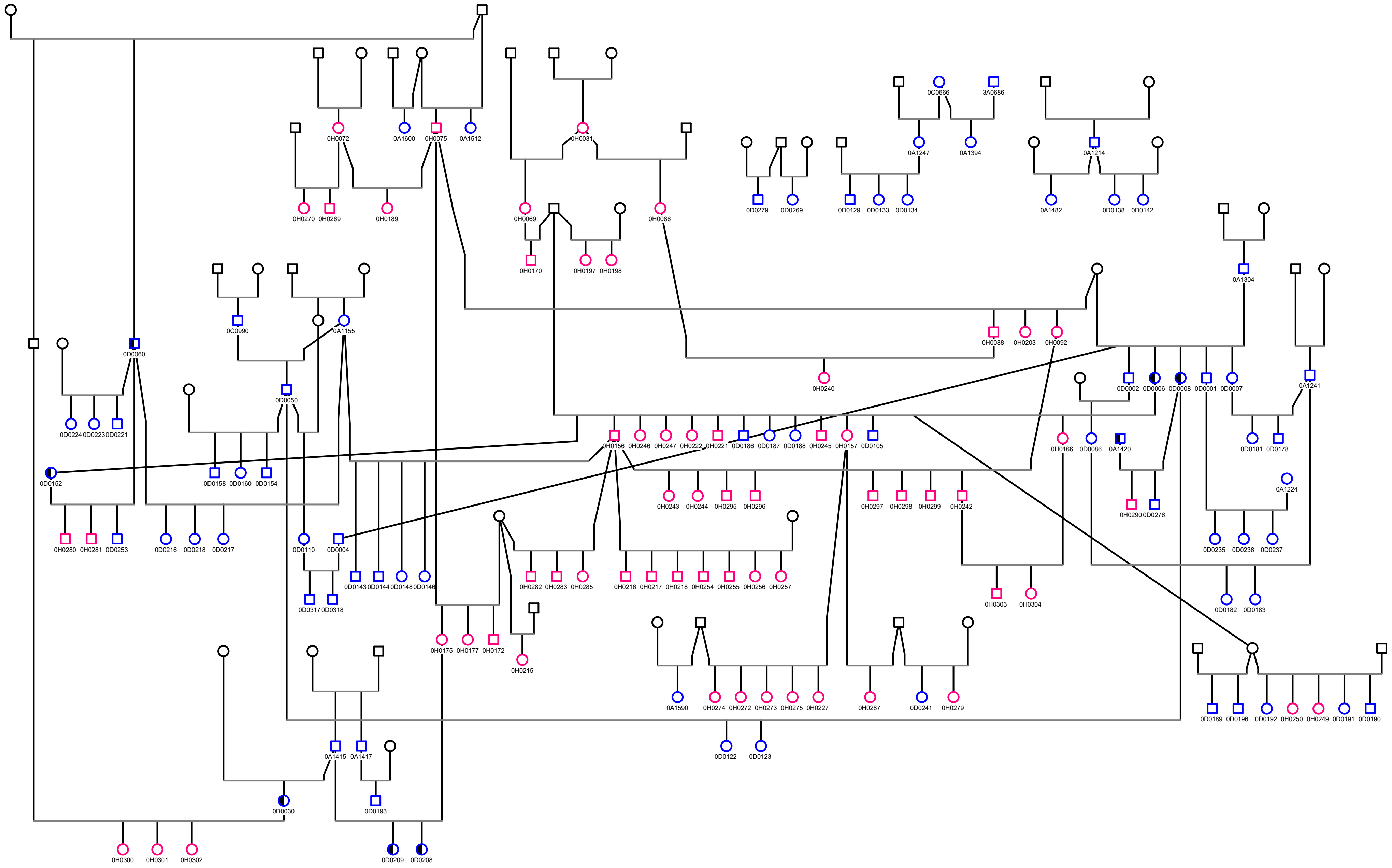

Figure S2. Pedigree data of 114 Sapsarees used in this study. The long-haired Sapsarees are shown in blue and the short-haired Sapsarees are shown in pink. Males and females are indicated by rectangles and circles, respectively. Possible heterozygotes are indicated as half-close symbols.

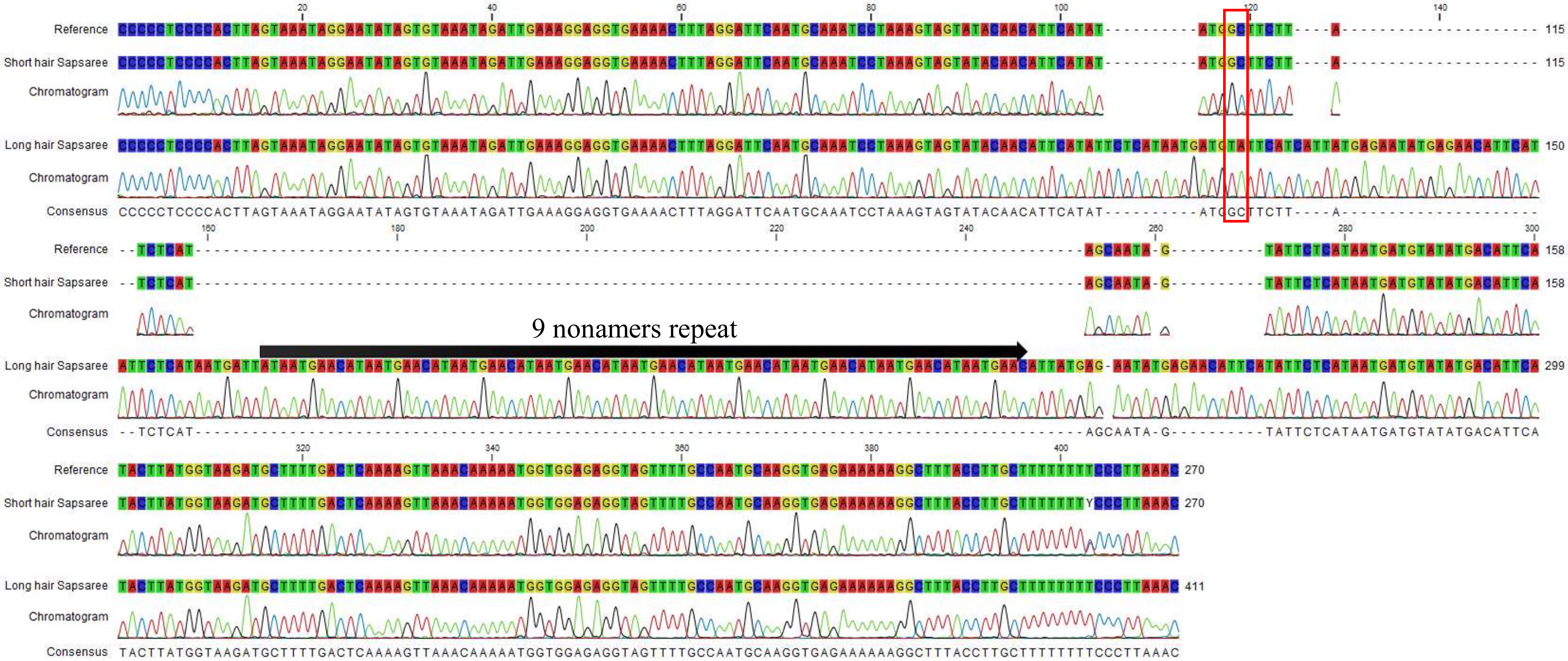

Figure S3. The nucleotide sequence alignment of the *RSPO2* 3′ UTR region between the long- and short-haired variants of Sapsarees. The partial *RSPO2* 3′ UTR sequence from the dog reference genome is shown on top. The sequencing results and associated chromatograms of the corresponding region are shown in the order of short and long hair alleles. The 9-nucleotide-repeat insertion in the long hair allele is indicated by the arrow. The two SNPs in the red box are specific for the two variants.

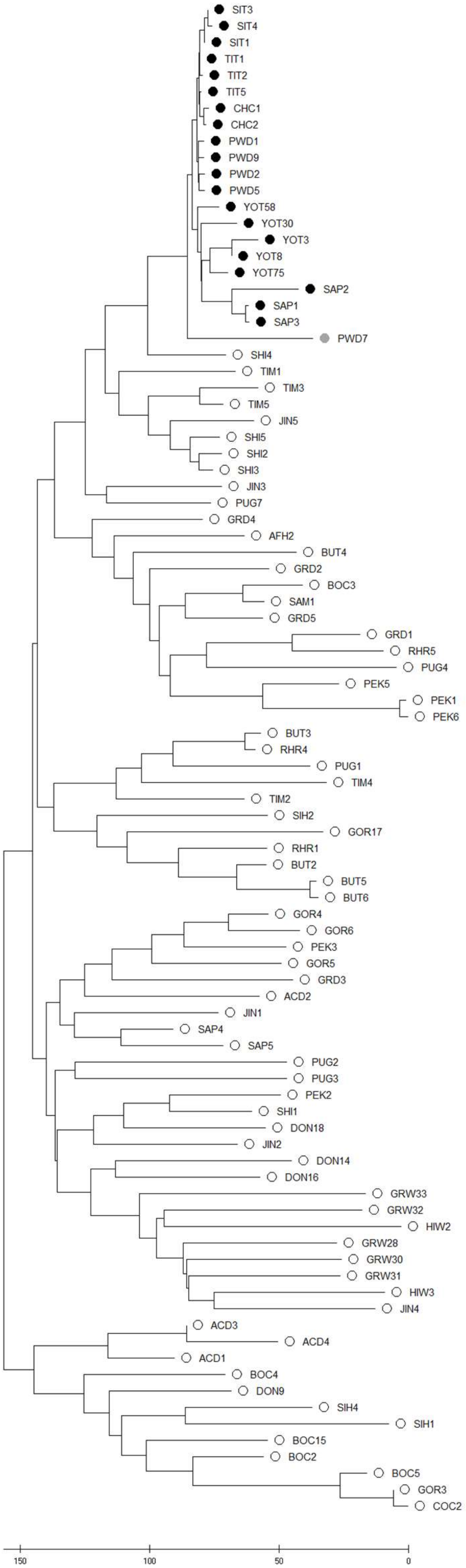

Figure S4. Neighbor-joining tree constructed using the CFA13:8,530,000–8,730,000 bp region of 92 dogs belonging to 24 dog breeds with various hair length phenotypes. Branch length was measured based on the number of nucleotide differences in the CFA13:8,530,000–8,730,000 bp region of each individual. Individuals homozygous for *RSPO2* 3' UTR insertion are indicated by black circles, and those without insertion are indicated by white circles. Gray circles indicate individuals heterozygous for the insertion. GOR (Golden Retriever), ACD (Australian Cattle Dog), AFH (Afghan Hound), YOT (Yorkshire Terrier), BOC (Border Collie), BUT (Bull Terrier), GRD (Great Dane), CHC (Chinese Crested), COC (Chow Chow), PWD (Portuguese Water Dog), RHR (Rhodesian Ridgeback), SIH (Siberian Husky), SAM (Samoyed), DON (Donggyengi), GRW (Grey Wolf), HIW (Himalayan Wolf), JIN (Jindo), PEK (Pekingese), PUG (Pug), SAP (Sapsaree), SHI (Shiba), SIT (Shih Tzu), TIM (Tibetan Mastiff), TIT (Tibetan Terrier).

(A)

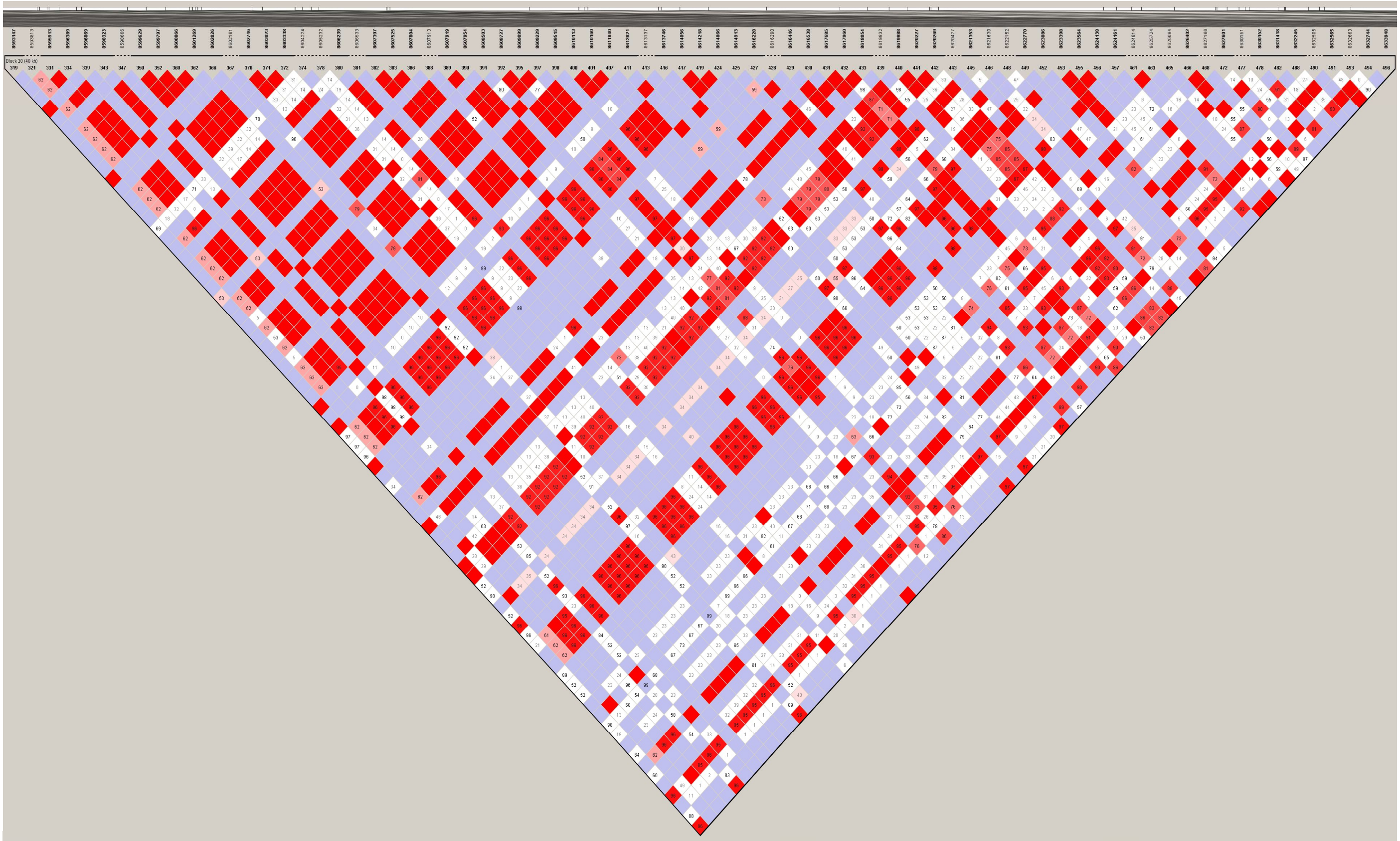

(B)

319 331 334 339 343 350 352 360 362 366 370 371 372 380 382 383 386 389 390 391 392 395 397 398 400 401 407 411 416 417 419 424 425 427 429 430 431 432 433 440 441 442 445 449 452 453 455 456 457 466 472 478 482 488 491 494 496

TGTAGTTAAGAAGCTTGCAAAGTGAGGGAATGTCTACGTGTGGAAACTCACTGCATC .298
*TGTAGTTAAGAAGCTTGCAAAGTGAGGGAATGTCTACGTATGGAAACTCGCTGCATC .266
TGTAGTTAAGAAGCTTGCAAAATGAGGGAATGTTCAAGCACCTATACTTGAGGTAGC .073
CGTGGTTAAAAAGCTTGCAAAGTGAGGAGATGCCTACATACCGGAACTCGCTGCGTT .069
TACACACGCGGGATCCATCAGACGGACGAGCATCTGCATACCGAAGTCCGCTACGTC .058
TGTAGTTAAGAAGCTTGCAGAGTAAGGGAATGTCTACGTATGGAAACTCGCTGCATC .028
TGTAGTTAAGAAGCTTGCAAAATGAGGGAATGTTCAAGCACCTAAACTTGAGGTAGC .025
TGTAGTTAAGAAGCTTGCAAAGTGAGGGAATGTCTACGTACCGGAACTCGCTGCATC .020
TGTAGTTAAGAAGCTTGCAGAGTAAGGGAATGTCTACGTATGGAAACTCACTGCATC .019
TGTAGTTAAGAAGCTTGCAAAGTGAGGAGATGCCTACATACCGGAACTCGCTGCGTT .016
TGTAGTTAAGAAGCTTGCAAAATGAGGGAATGTTCAAGCACCTAAACTTACTGCAGC .012
TGTAGTTAAGAAGCTTGCAAAATGAGGGAATGTTCAAGCACCTATACTTGCGGTAGC .011

Figure S5. Haplotype block 20 estimated by linkage disequilibrium of SNPs present in the *RSPO2* 3' UTR linked region on dog chromosome 13. (A) Plots were created based on the absolute value of LD (D') of the two markers. Each marker comparison was colored according to D' and the logarithm of odds (LOD) score; white for D' < 1 and LOD < 2, blue for D' = 1 and LOD < 2, pink for D' <1 and LOD ≥ 2, and red for D′ = 1 and LOD ≥ 2. Each block was designated as a marker comparison interval connected with 95% confidence of D' according to the definition of Gabriel et al. (2002). (B) Twelve major haplotypes with a frequency of 0.01 or higher are shown in the lower right. The haplotype related to the *RSPO2* 3' UTR insertion is separated by "*". The 11 tagging SNPs to differentiate haplotypes are indicated by "▼".

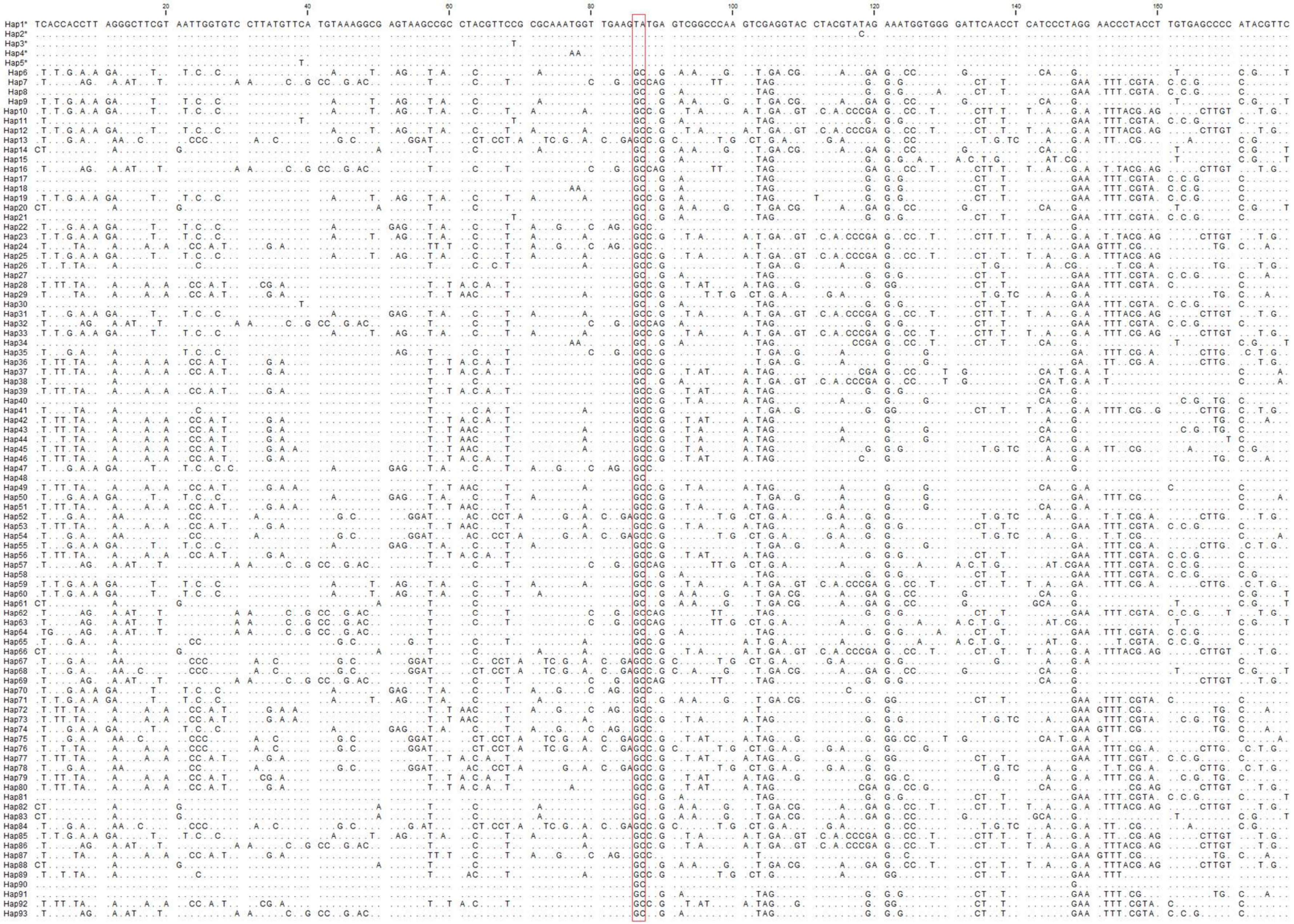

Figure S6. Result of multiple sequence alignment using 93 haplotype sequences of the *RSPO2* 3′ UTR selective sweep region. The first five haplotypes are associated with the *RSPO2*-repeat allele. SNPs identical to those of Hap1 are replaced by dots, and SNPs specific to the insertion-related haplotypes are indicated in a red box.

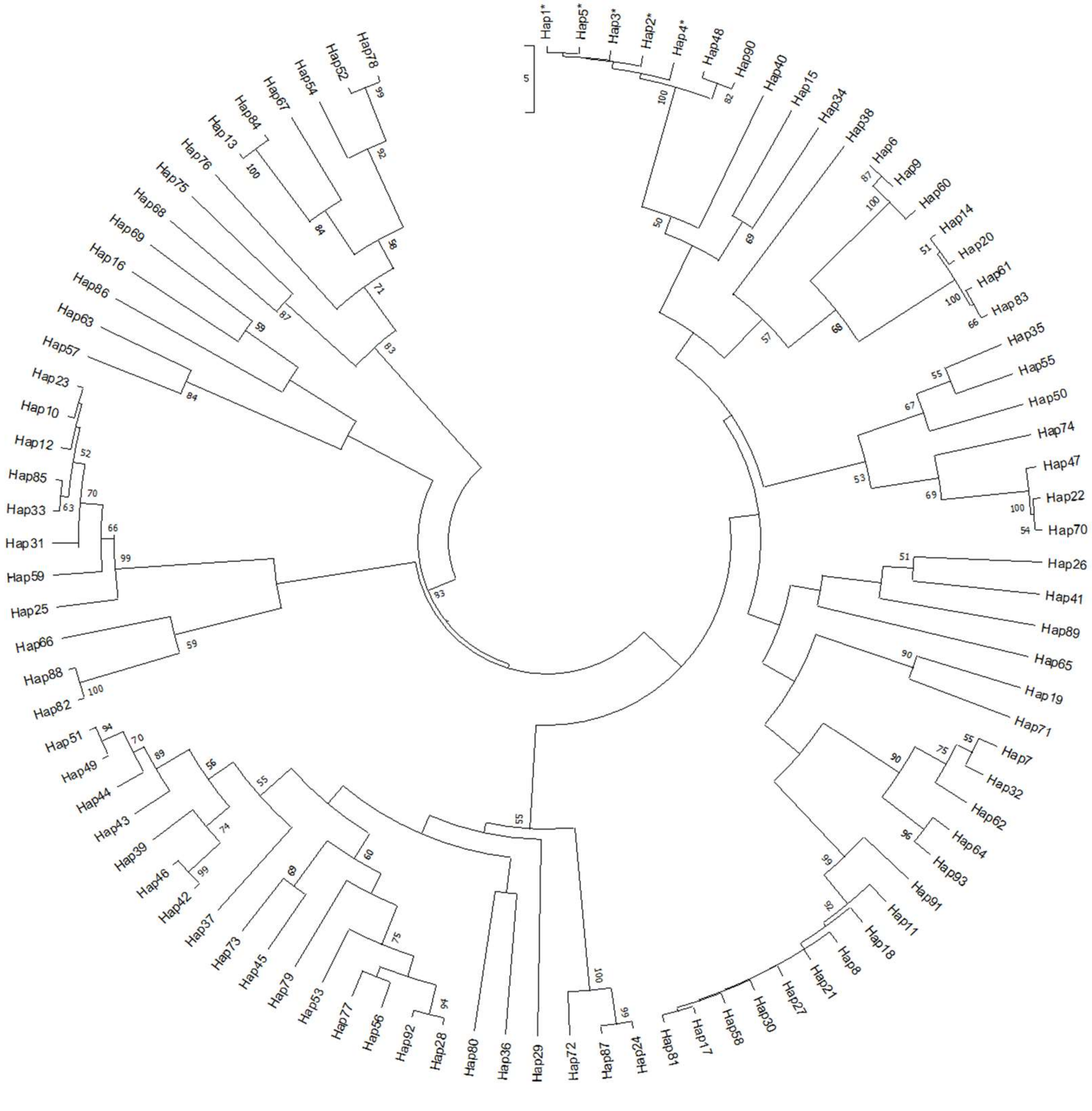

Figure S7. Phylogenetic relationships among the haplotypes of the haplotype block 20 region. A consensus UPGMA tree was created from the pairwise comparisons of 178 SNP markers present in 93 haplotypes with 1,000 bootstrapping replications. Branch length was determined using pairwise nucleotide difference. The 5 haplotypes associated with insertion are marked with asterisks.
